# Supplementary material for: Chromothripsis during telomere crisis is independent of NHEJ, and consistent with a replicative origin
Source: Genome Res. 2019 May;29(5):737–49. doi: 10.1101/gr.240705.118 (PMC6499312; doi:10.1101/gr.240705.118)
Supplement: Supplemental Material [file supp_gr.240705.118_Supplemental_file_1.zip › contigs/annotated_contigs/DB37/contig.2.DB37_length_408_mean_cov_5.61029411765.docx]

**DB37_length_408_mean_cov_5.61029411765**

```
TTTTAGAAAA|TAATAAAAAAAAAAATACAAAAAACTAAGATTGTAAAATTAAAAAA|AAA|AAAGAAAGCCAACTTTGTTTGAATTTA
           >chr5:56720732-56720778 + E=4e-07                >chr5:56721932-56722283 +
AAGTGGACCAGAGGGACAAATGGTGTGAAAAGCAGTGTGACTGATGATATCTAGTAGGTAGCAGATGCTGATGGAGGGAGGGAGGGTAG
E=4e-194
ACTGCATAGACAGGAAGGGCCTCAGTATAAGGTAAAAATGATTCATCATTAATAACGACTAATTTTTTTTAAGCATAGGTGTTGTGGTG

AAGGGACAAGTACAGGAGAAAGTATACAGTTATTTAATTATCTTTCCTAAATGTTGAAAACACACATGTAGCCTTTATAAGATATAGTA

TTTTGTGAATTAGATGAAGAGTTTGTGCATGGATACTGTGGTGCCTACTAGGGCA
```
